# Custom designed and 3D-printed titanium pelvic implants for acetabular reconstruction after tumour resection

**DOI:** 10.1177/11207000221135068

**Published:** 2022-11-21

**Authors:** Demien Broekhuis, Richard Boyle, Sascha Karunaratne, Alfred Chua, Paul Stalley

**Affiliations:** 1 Leiden University Medical Centre, Leiden, The Netherlands; 2 Department of Orthopaedic Surgery, Royal Prince Alfred Hospital, Camperdown, NSW, Australia; 3 Surgical Outcomes Research Centre (SOuRCe), Royal Prince Alfred Hospital, Camperdown, NSW, Australia; 4 Department of Anaesthetics, Royal Prince Alfred Hospital, Camperdown, NSW, Australia

**Keywords:** Custom, hemipelvectomy, oncologic resection, patient specific, reconstruction, short-term follow-up

## Abstract

**Background:**

Reconstructive procedure following resection of large pelvic tumours around the hip joint remains a complex challenge.

**Methods:**

This study presents a retrospective case series of patients presenting with benign or malignant pelvic tumour for which an internal hemipelvectomy including the hip joint and subsequent reconstruction with a custom designed 3-dimensional printed titanium pelvic implant (3DPPI) has been performed between August 2013 and January 2018.

**Results:**

15 consecutive patients with a median age of 33.9 years (IQR 26.4–72.2) and a median BMI of 20.7 kg/m^2^ (IQR 19.0–33.3) were reviewed after median follow-up of 33.8 months (IQR 24.0–78.1). The majority of patients presented with a malignant tumour as their principal diagnosis (*n* *=* 13, 86.7%). The median surgical time was 5.5 hours (IQR 4.5–8.5) and median peri-operative blood loss was 5000 ml (IQR 2000–10000). The median MSTS score at follow-up was 63.3% (IQR 51.7–86.7%). The median NRS in rest was 0.0 (IQR 0.0–5.0), the median NRS during activity was 2.0 (IQR 0.5–7.0) and the median HOOS-PS was 76.6% (IQR 67.9–91.0). 4 patients had implant-specific complications (*n* *=* 4, 26.6%); 1 hip dislocation (Henderson type 1a), 3 structural complications (type 3a), 1 deep infection (type 4a) and 1 local tumour recurrence (type 5b). At follow-up, 4 out of 15 implants were classified as a failure, resulting in an implant survival rate of 73.3%.

**Conclusions:**

Acceptable peri-operative outcomes, functional results, complication rates and short-term implant survival can be achieved in a cohort of complex patients undergoing 3DPPI reconstruction after hemipelvectomy including the acetabulum.

## Introduction

Reconstructive procedures following resection of large pelvic tumours, especially around the hip joint, remain a difficult and complex challenge. For primary bone tumour, and also increasingly for solitary metastatic pelvic tumour, clinicians are constantly trying to improve reconstruction techniques. The most important aims focus on longevity of the reconstruction and improving functional outcomes, without conceding tumour resection margins.

**Figure 1.**
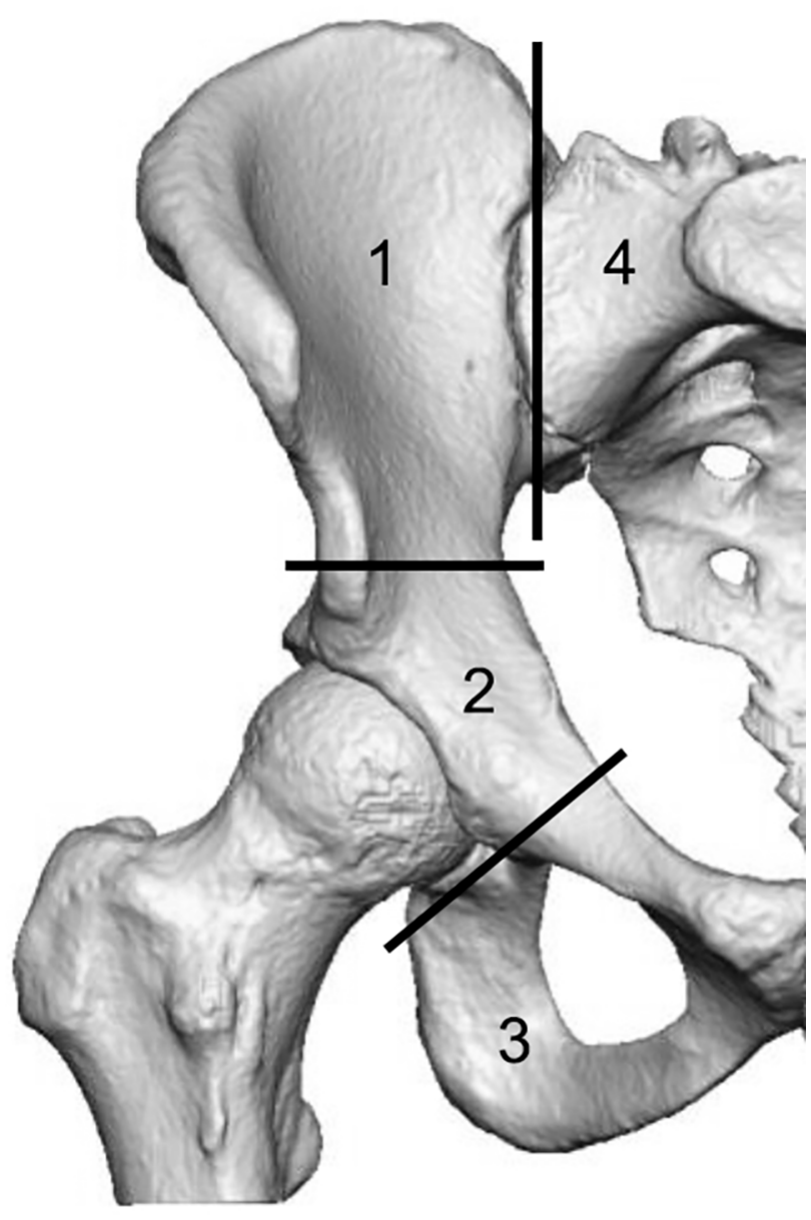
Enneking’s classification of pelvic resections (1); P1-ilium; P2-peri-acetabulum; P3-pubis; and P4-sacrum.

Pelvic resections can be categorised using the Enneking classification (Figure 1).^1^ Endoprosthetic reconstruction is the preferred technique for pelvic resections including the acetabulum (P2), although the most optimal reconstructive method has been widely debated.^2,3^ Endoprosthetic reconstruction options include saddle endoprosthesis,^4,5^ pedestal cup endoprosthesis,^6–8^ modular endoprosthesis^9–12^ and custom-made implants.^13–18^ Bio-logical reconstruction techniques include extra-corporeal irradiation and re-implantation (ECI),^19–22^ resection hip arthroplasty^23,24^ or iliofemoral arthrodesis.^25^ The risks of mechanical and non-mechanical complications associated with all these techniques are high. Saddle endoprosthesis relay heavily on remaining iliac bone (P1) and due to associated high risk of complications and poor long-term functional outcomes,^4^ this technique has been superseded by other endoprosthetic solutions. Pedestal/ice-cream cone endoprosthesis rely on remaining posterior iliac bone for fixation. The difficulties and limits of pedestal stem positioning in the remaining posterior pelvis may lead to altered hip centre of rotation, and high incidences of hip dislocation have been reported.^6^ Modular pelvic implants have the downside that due to the non-custom fit, fixation to the remaining pelvis can be limited and high rates of short-term aseptic loosening have been reported.^9^ Finally, early generations of patient specific custom-made endoprosthesis were associated with high incidences of implant aseptic loosening, likely attributed to limited implant fixation options and the lack of long term osseointegration.^14^

The improvements in rapid prototyping and 3-dimensional metal printing technology have enabled custom designed 3-dimensional printed titanium pelvic implants (3DPPI) to become clinically available over the last decade. The evidence on 3DPPI in the setting of pelvic tumour resections including the P2 area is still limited to case reports and small case series.^13,17,18,26–29^

The aim of this study was to evaluate the clinical and functional performance of 3DPPI for pelvic reconstruction including the P2 region since its introduction at our institution in 2013. The main outcomes evaluated were the clinical and functional results, implant specific complications and implant survival. We have also included a brief description on the specifics of the 3DPPI design, manufacturing process and surgical technique.

## Patients(/materials) and methods

This study was designed in accordance with the STROBE (STrengthening the Reporting of OBservational studies in Epidemiology) statement guidelines.^30^ The Human Research Ethics Committee of the Royal Prince Alfred Hospital, Sydney, Australia (HREC Number LNR/17/RPAH/423) approved the study. We conducted a retrospective cohort study of all consecutive patients undergoing acetabular reconstruction with a 3DPPI between January 2013 and January 2018 in our tertiary oncological clinic in Sydney, Australia. Patients who had undergone hemipelvectomy including the P2 acetabular area for benign or malignant (primary or metastatic) tumours were included. Patients in which a 3DPPI was used for revision total hip arthroplasty (THA) or other indications were excluded. During the studied period, no other types of acetabular endoprosthetic tumour implants (e.g. pedestal cups) were used. The magnitude of bone destruction, patient prognosis and expected morbidity were used to assess if patients with metastatic disease were indicated for hemipelvectomy and 3DPPI reconstruction.

Data were retrieved from patient hospital medical records. Functional outcome scores obtained included the Musculoskeletal Tumour Society score (MSTS), the Numeric Rating Scale (NRS) for pain in rest and activity and the Hip disability and Osteoarthritis Outcome Score-Physical Function Short Form (HOOS-PS). Implant specific complications were categorised using the Henderson tumour prosthesis complication classification.^31^ Survival of the prosthesis was defined using revision of the 3DPPI for any reason (e.g. complete revision, unplanned revision of a failed portion, fixation of a periprosthetic fracture, soft-tissue reconstruction to restore joint stability, endoprosthetic removal without revision, and amputation) as the endpoint.

In the studied period, a total of 24 patients underwent 3DPPI for reconstruction for pelvic defects including the P2 area. 9 patients were excluded: 8 for total hip arthroplasty revision indication and 1 for chronic congenital hip dislocation. The final analysis included 15 patients (9 males, 6 females) with a median age of 33.9 years (interquartile range [IQR] 26.4–72.2) and a median body mass index (BMI) of 20.7 kg/m^2^ (IQR 19.0–33.3) (Table 1). The median follow-up period was 33.8 months (IQR 24.0–78.1), with no patients lost to follow-up.

**Table 1.** Demographics, peri-operative data, complications and functional scores of patients reconstructed with a 3DPPI (*n* = 15).

Patient	Sex	Age (years)	BMI (kg/m^2^)	Follow-up (months)	Histological diagnosis	Malignant/Benign (M/B)	(Neo-adjuvant) radiotherapy	Enneking type resection	Histological margins	Surgical time (hours)	Blood loss (ml)	Henderson	Implant related complications (treatment)	Disease status	MSTS	HOOS-PS	NRS rest	NRS activity
						Primary/Secondary (POB/POS/SIB*)						Complication		DFS/AWD/DOD**				
						Metastatic disease at presentation (Met)						Classification						
1	m	67.1	20.2	2.7	Renal cell carcinoma	M/SIB	y	2 + 3	Clear	6	9000			DOD	na	na	na	na
2	f	40.5	19.3	78.1	Osteosarcoma	M/POB	n	1 + 2	Clear	2.5	1500			DFS	87%	80%	0	0
3	m	72.2	25.4	34.4	Chondrosarcoma	M/POB	n	2	Clear	4.5	2000			DOD	na	na	na	na
4	m	71.8	26.6	36.3	Renal cell carcinoma	M/SIB	n	1 + 2	Clear	4.5	6000			DFS	57%	80%	0	1
5	m	20.6	17.0	59.7	Ewing sarcoma	M/POB	y	1 + 2	Clear	8	10,000			DFS	83%	91%	0	0
6	m	52.1	33.3	34.7	Renal cell carcinoma	M/SIB	n	2 + 3	Clear	8.5	7000	3a. 4a	- Loose pubic screws (replacement) -chronic infection (supressive antibiotics)	DFS	27%	44%	5	2
7	m	29.0	27.3	33.8	Chondrosarcoma	M/POB	n	2 + 3	Clear	8.5	10,000	1a	-Hip dislocation (open reduction)	DFS	63%	77%	0	3
8	f	31.0	21.2	23.9	Breast carcinoma	M/SIB/Met	y	1 + 2 + 3	Positive	5.5	6500	3a. 3a	-Prominent flange (trimming) -Srew S1 radiculopathy (replacement)	DOD	na	na	na	na
9	f	23.7	19.5	25.5	Neurofibroma	B/POS	n	2	Clear	3.5	1400			DFS	87%	80%	0	0
10	f	31.1	17.8	24.4	Osteosarcoma	M/POB	n	1 + 2 + 3	Clear	4.5	2400			DFS	47%	70%	0	2
11	m	40.2	20.7	17.0	Chondrosarcoma	M/POB	n	1 + 2 + 3	Clear	5.5	4000	5b	-Tumour recurrence (hindquarter amputation)	DOD	na	na	na	na
12	m	33.9	26.1	30.4	Testicular carcinoma	M/SIB	n	1 + 2	Clear	6	2000			DFS	63%	73%	5	7
13	m	38.0	30.7	27.3	Giant cell tumour of bone	B/POB	n	2	Clear	5	na			DFS	43%	66%	3	7
14	f	22.6	18.6	45.7	Ewing sarcoma	M/POB	n	1 + 2	Clear	3	1400			DFS	63%	58%	0	5
15	f	16.0	18.6	44.0	Osteosarcoma	M/POB	n	2	Clear	4.5	na			DFS	67%	70%	0	3
Median		33.9	20.7	33.8						5.5	5000				63%	77%	0	2

* POB, primary of bone; POS, primary of soft tissue; SIB, secondary in bone.

** DFS, disease free survival; AWD, alive with disease; DOD, died of disease.

The main indication for surgery in our cohort was malignancy (*n* *=* 13, 86.7%), where 8 patients had a primary bone malignancy (3 chondrosarcoma, 3 osteosarcoma and 2 Ewing sarcoma) and 5 patients had a pelvic metastatic lesion (3 renal cell, 1 breast and 1 testicular carcinoma). There were 2 benign lesions (1 neurofibroma and 1 giant cell tumour of bone) (Table 1). In these 2 patients, the extent of bone destruction and management of the benign lesions was comparable to a P2 hemipelvectomy, and reconstruction could not be managed with regular or modified total hip arthroplasty techniques, hence the choice for 3DPPI reconstruction. Clear resection margins were achieved in 12 of the 13 malignant cases (92%) and in both benign patients. A total of 5 patients died of disease; 2 patients with metastatic disease from bone primary whilst 3 patients died due to known distant primary carcinoma (2 renal-cell and 1 breast carcinoma). 1 patient (breast carcinoma metastasis) was known to have lung metastasis at time of surgery (Table 1).

### Design and manufacturing process

**Figure 2.**
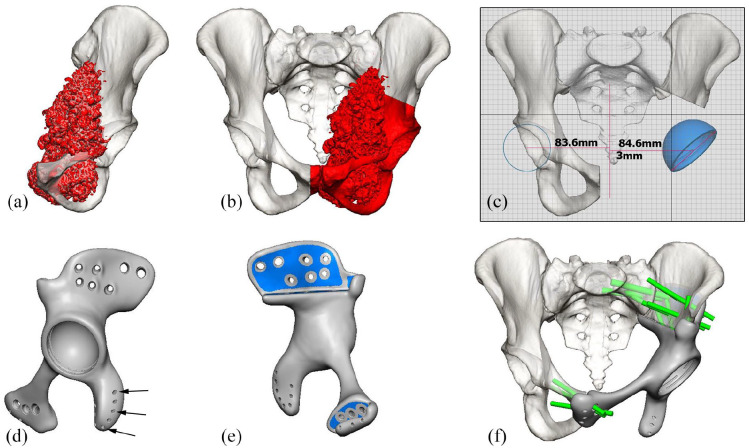
3D computer renders for the design of a 3DPPI for a patient with a left sided pelvic chondrosarcoma: (a) frontal view of the left hemi-pelvis, with the chondrosarcoma highlighted [red], (b) frontal view of pelvis with the planned P2-3 resection highlighted [red], including a part of the contralateral os pubis, (c) frontal view of the pelvis with the planned centre of hip rotation for the 3DPPI, based on the contralateral side, (d) lateral view of the completed 3DPPI design including suture holes for soft tissue reattachment (arrows), (e) medial view of the 3DPI design, indicating the porous surface areas highlighted [blue], and (f) frontal view of the pelvis with the 3DPPI in place and locking head screw locations and trajectories highlighted in green. Images used with permission of OSSIS Limited, Christchurch, New Zealand, all rights reserved.

**Figure 3.**
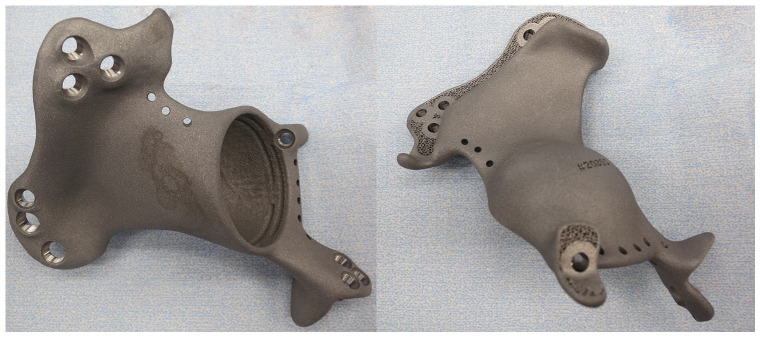
A 3DPPI used for acetabular reconstruction after resection of a P2 metastatic breast carcinoma lesion.

The custom-made implant was designed in close collaboration with the surgeon and manufactured by OSSIS Limited (Christchurch, New Zealand), using data from computed tomography (CT) scan of individual patient pelvis in 0.625–1.25 mm slices. Image segmentation and subtraction analysis was used to create a three-dimensional model of the relevant anatomy (Figure 2(a)). Tumour resection and osteotomy sites were specified by the surgeon (Figure 2(b)). Contralateral centre of hip rotation (Figure 2(c)), bone anatomy and force transference were taken into consideration during the design process (Figure 2(d)). The implant was designed to close the pelvic ring, with fixation points to the remaining bone on at least 1 superior (ilium of sacrum) and 1 or 2 inferior flanges (ramus inferior, ramus superior or contralateral pubis). Porous surfaces at bony interfaces were added (Figure 2(e)). The pores had an average size of 658 μm, averaging a porosity of 73%. Multiple 5.0- and 7.3-mm titanium alloy locking screw holes were incorporated in the flanges with pre-planned lengths and trajectories according to CT bone quality, expected implant loading and surgical approach (Figure 2(f)). The final implant was 3D printed by additive manufacturing process with electron beam melting (EBM) using Ti6Al4V alloy powder (Figure 3). In addition, a plastic stereolithography printed trial implant, pelvic bone model and patient specific cutting guides were provided for intra-operative use. The time required for design and manufacture was approximately 2–6 weeks.

### Surgical technique

**Figure 4.**
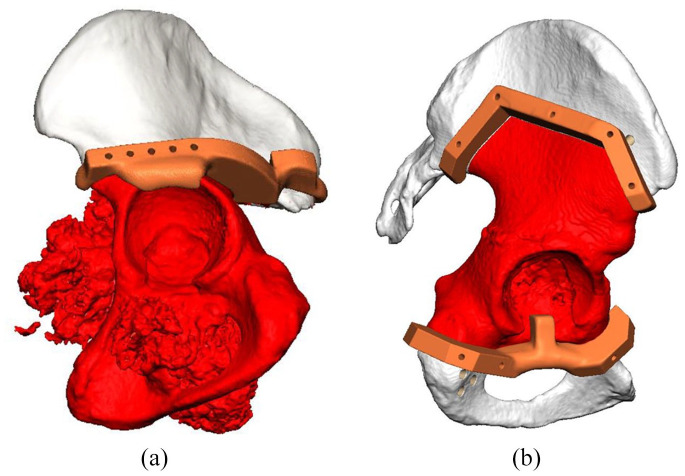
3D computer renders of two patients, including the planned resection highlighted in red and the plastic 3D printed patient specific cutting guides highlighted in orange: (a) lateral view of a pelvis of a patient treated for chondrosarcoma and (b) lateral view of a pelvis of a patient treated for metastatic breast carcinoma. Images used with permission of OSSIS Limited, Christchurch, New Zealand, all rights reserved.

**Figure 5.**
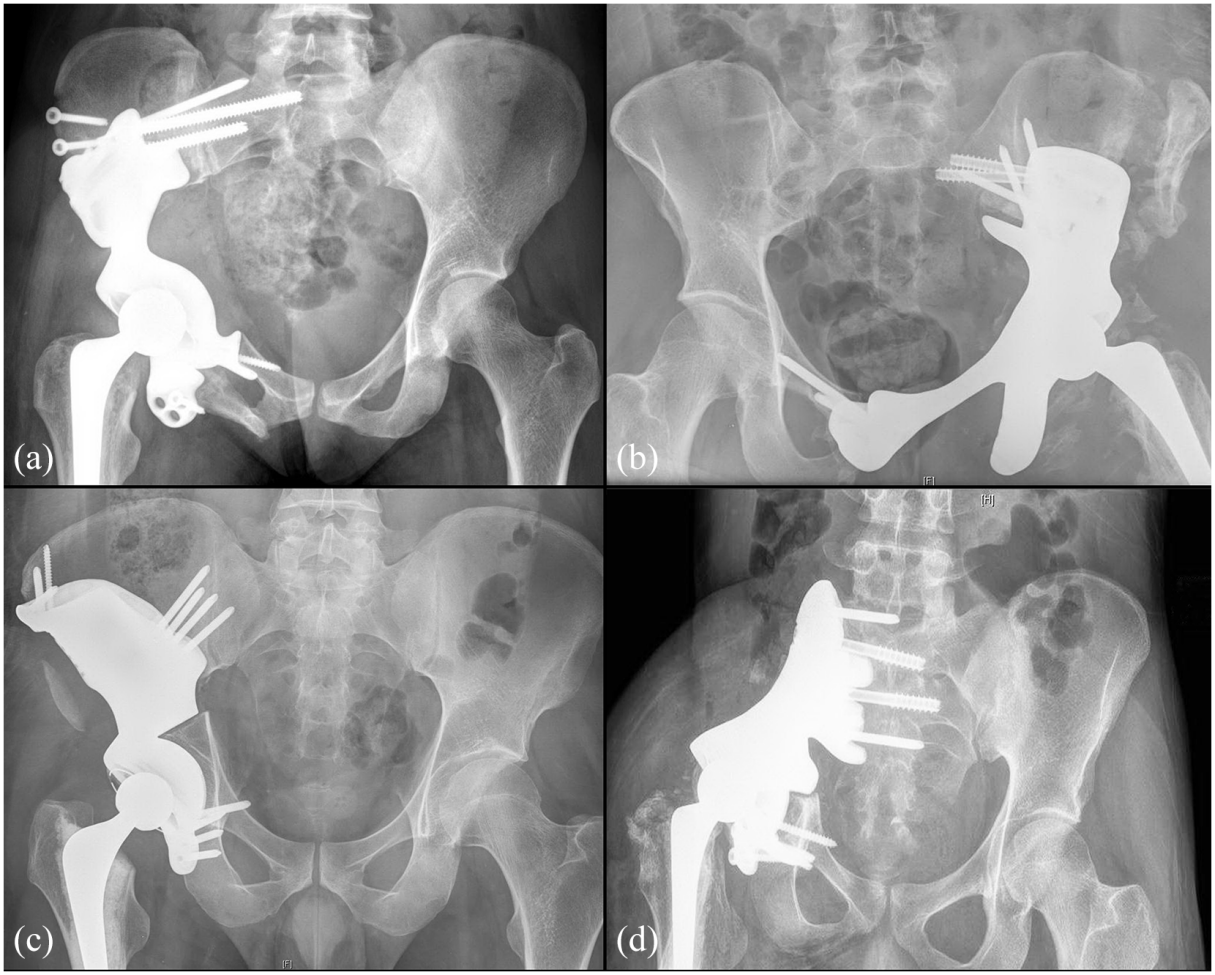
Four post-operative antero-posterior x-rays of a pelvis showing the implantation of a 3DPPI for (a) P1-2-3 resection for metastatic breast carcinoma, (b) P2-3 resection for chondrosarcoma, (c) P1-2 resection for metastatic testicular carcinoma, and (d) P1-2 resection for recurrent Ewing sarcoma.

All the operations were performed by 1 of 2 senior authors of this paper (RB/PS). The procedures were carried out via a modified iliofemoral approach with or without anterior superior iliac spine osteotomy for abductor release. Patient specific cutting guides were used to facilitate osteotomies (Figure 4). The plastic trial implant was used to approximate implant positioning. The 3DPPI was secured to the remaining bone by locking screws, using drilling guides that provided all the pre-determined screw trajectories. The femoral side was reconstructed with a cemented femoral stem or a proximal femoral replacement (Exeter or Global Modular Replacement System, Stryker, Mahwah, NJ, USA). On the acetabular side, a semi-constrained polyethylene Snap-Fit cup (Bioimpianti, Milan, Italy) was cemented into the 3DPPI acetabular dome with antibiotic (gentamycin) loaded polymethylmethacrylate cement. The anterior superior iliac spine osteotomy was re-attached with 4.5 mm large fragment screws. Wounds were closed over suction drains. All the patients were admitted to the intensive care unit. They received 6 weeks thromboprophylaxis (low molecular weight heparin) and antibiotics in accordance with our institutional protocol. Patients remained on bed rest until muscle control was regained. Rehabilitation followed under physiotherapist supervision and guidance. Figure 5 shows a postoperative x-ray of 4 patients treated with a 3DPPI.

### Statistical analysis

Statistical Package for the Social Science (SPSS) software version 23.0 (SPSS Inc., Chicago, IL, USA) was used to perform the statistical analysis. Nominal data was analysed as total number per group with percentages of the total. Numeric data was calculated using the median and its variability presented as the interquartile range (IQR).

## Results

### Clinical and functional outcomes

The median surgical time was 5.5 hours (IQR 4.5–8.5) and median estimated blood loss was 5000 ml (IQR 2000–10000). The median length of stay in hospital was 16.0 days (IQR 16.0–73.0). Patients remained bed rest for a median 8.5 days (IQR 7.0–21.0) and commenced full weight-bearing after a median of 6.0 weeks (IQR 6.0–26.0). Functional scores were obtained at the last alive time of follow-up. The median MSTS score was 63.3% (IQR 51.7–86.7%), the median NRS rest was 0.0 (IQR 0.0–5.0), the median NRS activity was 2.0 (IQR 0.5–7.0) and the median HOOS-PS (where 100% represents no limitations) was 76.6% (IQR 67.9–91.0%) (Table 1).

### Complications and implant survival

**Figure 6.**
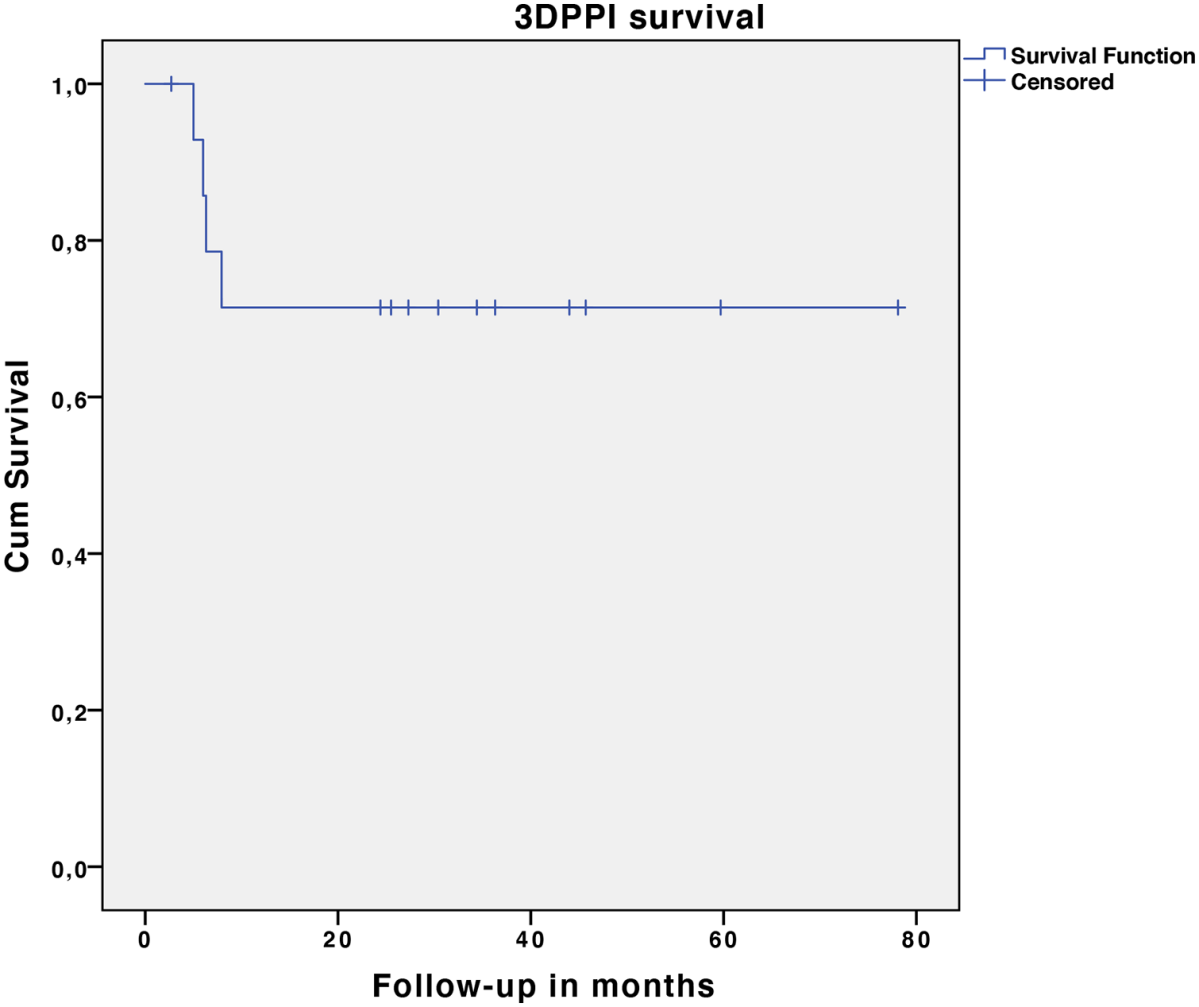
Kaplan-Meier plot of the 3DPPI survival analysis.

4 patients had implant specific complications (n = 4, 26.6%). These comprised of 1 hip dislocation (Henderson type 1a), 3 structural complications (type 3a), 1 deep infection (type 4a) and 1 local tumour recurrence (type 5b). There were no incidences of aseptic loosening (type 2) recorded. The hip dislocation occurred 8 weeks after surgery and was managed with open reduction following debridement of peri-articular ossifications. There was no further dislocation in this patient at 34 months follow-up. 1 patient presented with symptomatic loosening of 2 superior ramus flange locking screws, without any radiological evidence of implant loosening, 6 months after surgery and was managed with replacement of the loose screws. The same patient was diagnosed with a chronic low-grade infection, 34 months after implantation. Radiographic evidence of osteolysis around the pubic flange screws, without clear loss of fixation/migration of the implant at the ilium was observed. Surgically obtained cultures were positive and management at last follow-up consisted of suppressive antibiotics with implant retainment. 1 patient presented with a symptomatic prominent pubic ramus flange, which resolved after surgical trimming of this flange. The same patient also suffered from S1 radiculopathy, which was related to a screw compromising the S1 neuroforamen, with exchange to a shorter screw providing immediate relief of symptoms. 1 patient treated for chondrosarcoma was diagnosed with periprosthetic tumour recurrence 5 months after surgery, resulting in a hindquarter amputation. This patient deceased 12 months later from metastatic disease. 4 out of 15 implants were classified as an implant failure, resulting in an implant survival rate of 73.3% (Figure 6).

## Discussion

There have been very few studies assessing the use of custom 3D printed pelvic implants for tumour hemipelvectomy.^17,18,32^ In our study, we specifically aimed to evaluate this technique for reconstruction after hemipelvectomy including the acetabulum. To our knowledge, this study is one of the largest 3DPPI series to date in this specific cohort of tumour patients. We believe that, based on our short-term outcomes and when compared to international literature, this technique shows satisfactory clinical results, functional outcomes, complication rates and survival.

One of the biggest advantages of this technique over existing alternatives is the individualised nature of each implant relative to the patient anatomy and tumour configuration, allowing accurate hip reconstruction. Compared to early generation custom pelvic implants, 3DPPI incorporate an increased number of large (locking) screws for short-term fixation, porous and/or hydroxyapatite bone surfaces for long-term osseointegration and an anatomic design to reconstruct the hip centre of rotation. Patient specific screw, drill and osteotomy guides and sterile bone models for intra-operative use are often available and are intended to improve surgical ease and accuracy. Furthermore, 3DPPI have allowed for fixation to the sacrum after complete ilium resections (through or even medial to the sacroiliac joint), making this a universal technique suitable for essentially all pelvic locations (P1–P4).

The median MSTS score in our study was 63.3%. This is comparable with studies on new generation custom implants, that reported mean MSTS scores ranging between 66% and 77%.^13,17,18^ Most of the other reconstruction endoprosthesis techniques, such as pedestal cups and non-custom modular implants, have reported comparable or slightly lower MSTS scores, ranging between 49% and 70%.^6,9,12,33–36^ Older generations custom implants appeared to produce lower scores, between 37% and 70%, which might be related to high complication and aseptic loosening rates.^14–16,37–40^ Table 2 provides a summary of the most important results of endoprosthetic pelvic reconstruction techniques following hemipelvectomy including the acetabulum in current literature.

**Table 2.** Literature overview of papers reporting on endoprosthetic acetabular reconstruction techniques after tumour resection.

Author	Number of patients	Follow-up (months)	Type of implant	Pelvic ring restoration	Bloodloss (ml)	Surgical time (hours)	Clear margins	Dislocation	Aseptic loosening	Structural failure	Infection	Local recurrence	Implant survival	MSTS
Pelvic tumour implants non custom or modular														
Bus et al.^33^	19	39	Pedestal zimmer	No	na	na	na	26%	15%	21%	47%	21%	50%	49%
Bus et al.^6^	47	47	LUMiC implantcast	No	2300	6.5	87%	21%	1%	6%	30%	11%	82%	70%
Ji et al.^34^	100	53	Modular	na	2700	na	91%	9%	2%	5%	15%	20%	64%	57%
Wang et al.^35^	50	54	Modular	Yes	4200	6.8	82%	4%	2%	10%	14%	18%	64%	61%
Menendez et al.^36^	24	29	Modular	No	na	na	na	12%	na	8%	32%	20%	84%	67%
Guo et al.^9^	28	30	3D printed modular	Yes	4800	5.4	96%	4%	0%	7%	14%	25%	93%	62%
Liang et al.^12^	35	21	3D printed modular	No	2206	4.3	83%	6%	0%	0%	0%%	14%	100%	64%
Pelvic tumour implants custom. old generation														
Witte et al.^37^	40	24	Mutars	No	na	na	98%	3%	8%	15%	30%	18%	61%	50%
Jaiswal et al.^14^	98	65	Stanmore	No	na	na	na	20%	24%	3%	30%	31%	68%	59%
Abudu et al.^15^	35	84	Stanmore	No	na	na	na	17%	3%	6%	26%	23%	na	70%
Müller et al.^38^	9	62	Howmedica	Yes	na	na	na	11%	22%	na	56%	0%	67%	na
Ozaki et al.^16^	12	57	Howmedica	Yes	na	na	92%	8%	8%	33%	25%	50%	58%	37%
Tunn et al.^39^	24	98	Mutars	Yes/No	2300	8.8	96%	4%	17%	na	42%	21%	33%	na
Dai et al.^40^	10	34	Custom	Yes	na	na	na	20%	0%	10%	30%	34%	100%	na
Pelvic tumour implants custom. new generation														
Wang et al.^13^	13	27	Chunli Co	Yes	2600	4.3	na	0%	0%	0%	15%	0%	100%	77%
Peng et al.^17^	6 (5 oncology)	30	Custom	Yes	2500	4.1	na	0%	0%	0%	0%	0%	100%	66%
Angelini et al.^18^	41 (22 oncology)	60	Multiple manufacturers	na	1200	4.5	76%	0%	0%	0%	9%	0%	100%	70%
*This study*	15	34	OSSIS	Yes	5000	5.5	92%	7%	0%	20%	7%	7%	73%	63%

Patients in our study reported good HOOS-PS scores (median 76.6%), which is in line with our observed MSTS scores. While the HOOS-PS has not been specifically validated for patients receiving pelvic tumour endoprosthesis, it is commonly used to assess patient’s hip function during daily activities. This patient-reported tool was employed to balance the use of the MSTS, which is clinician-reported and often found to overestimate the ability of patients.^41^

In our cohort, clear oncological resection margins were acieved in 92% of cases, which is consistent with 82–98% clear margins reported in the literature.^6,12,16,18,34,35,37,39^ This result demonstrates that the use of patient specific cutting guides did not compromise surgical margins in our cohort. It also indicates there is no evident negative effect on radicality caused by the potential longer intervals between diagnosis and surgery (2–6 weeks to manufacture 3DPPIs) compared to the non-custom alternatives which are usually available within a shorter timeframe.

The use of custom-made implants in pelvic oncological surgery has been described in reports dating back to 1997. However, most publications were related to older generation custom implants. These implants range from hand crafted titanium implants attached to the bone with pins and cement, to more anatomically shaped implants with conventional screws. Significant rates of implant specific complications were recorded, with up to 20% dislocations, 24% aseptic loosening, 33% structural complications, 56% infections, and 50% local recurrences.^14–16,37–40^ More recently in 2020, Wang et al.^13^ published their series of 13 patients treated with custom 3D-printed titanium pelvic implant after tumour resection including the acetabulum. This newer generation implant technique incorporated pelvic ring reconstruction, porous surfaces and locking screws for fixation. At a mean of 27 months follow-up, they found 15% infections, but reported no dislocations, aseptic loosening, structural failures or local recurrences. Our results were in line with these results, on a similar sized group with a comparable follow-up period and type of implant.

Reports on non-custom pelvic implants (e.g. pedestal cups or modular endoprostheses) have reported higher rates of dislocations (4–26%), aseptic loosening (0–16%), structural complications (5–21%), infections (14–47%) and local recurrences (11–25%).^6,9,12,33–36^ Nevertheless, it must be noted that cohorts in these series are often larger, and reported follow-up often longer. A systematic review focussing on reconstructive techniques after periacetabular oncological resections by Brown et al.^3^ in 2018 also reported high numbers of complications for all types of endoprosthetic techniques, demonstrating the complexity of this patient cohort.

We acknowledge that our reported structural complication rate is relatively high, which might be a reflection of a learning curve during the introduction of this technique. Nevertheless, observed complications could be managed with relatively simple surgical interventions. Ongoing experience with this technique in our clinic and evolving implant designs have resulted in reduced implant-specific complications in more recent years.

Careful patient selection is paramount in utilising this implant, and the indication for the use a 3DPPI in patients with metastatic pelvic lesions should be reticent. Although patient numbers are low, our data showed that complications and death within 2 years after implantation occurred more often in patients treated for a metastatic pelvic lesion. It can be argued that, if in any means possible, only non-operative palliative therapy (e.g. radiotherapy/systemic therapy) could be considered as an alternative in non-curative patients, also from a cost-benefit perspective. Nevertheless, continuous improvement in survivorship of disseminated patients due to advancement in oncological systemic therapies and decreasing cost of 3DPPI’s over time might shift the balance even more towards long term solutions such as 3DPPI.^42^

We recognise that our study has several limitations. Firstly, although meticulous chart reviews and complete data accumulation has been pursued, the retrospective design of this work poses the largest limitation. Secondly, although our study reported on one of the largest groups of 3DPPI reconstructions for oncologic pelvic resection including the P2 area, the cohort was still small. Thirdly, there were no pre-operative functional scores available, and functional scores were not obtained at similar follow-up intervals, making it impossible to assess the early functional outcome and its improvement over time. In adition, 5 patients had deceased within the study period and therefore we were not able to assess their functional scores. Also, the 3DPPI technique itself has potential draw-backs. In order to achieve the desired implant fit, accurately produced osteotomies that are identical to the pre-operative plan are necessary. Intra-operative positioning of the patient specific cutting guides can be difficult and guides might not always fit, due to soft tissue interference and/or a mismatch between bone surface and guide contours. Understandably, extensive surgeon experience is crucial in order to achieve good outcomes with this technically demanding technique. Another draw-back is the relative high implant cost compared to the non-custom counterparts. Finally, the design, production and thereby surgical availability of custom produced implants for tumour indications need to be fast, and a prolonged lead-time can be a significant disadvantage in this patient group.

There are a several areas in this reconstructive technique that could be the focus of future research. In terms of long-term implant survival, model based Radio Steriometric Analysis (mRSA) can be of great value to identify early migration patterns in order to predict long-term implant fixation, as already demonstrated in primary total hip arthroplasty RSA studies.^43,44^ Furthermore, the custom shape of the implant necessitates precise osteotomies to achieve ideal implant position and bone apposition. Although the use of patient-specific cutting guides, in our experience, was accurate and beneficial, osteotomy adjustments were required in some instances before adequate implant positioning was achieved. Post-operative assessment of osteotomies and implant positioning in relation to pre-operative plan could validate accuracy of this technuque. Finally, studying the effect of intra-operative navigation in order to improve osteotomy accuracy, verify intra-operative implant positioning and screw trajectories could be of value.

## Conclusion

This study has shown that acceptable peri-operative outcomes, complication rates, functional results and short-term implant survival can be achieved in a cohort of complex oncological patients undergoing 3DPPI reconstruction after internal hemipelvectomy including the acetabulum. However, longer follow-up is important to assess long-term outcomes.

## References

[1] Enneking WF Dunham WK . Resection and reconstruction for primary neoplasms involving the innominate bone. J Bone Joint Surg Am 1978; 60: 731–746. 701308

[2] Angelini A Calabrò T Pala E , et al. Resection and reconstruction of pelvic bone tumors. Orthopedics 2015; 38: 87–93. 25665107 10.3928/01477447-20150204-51

[3] Brown TS Salib CG Rose PS , et al. Reconstruction of the hip after resection of periacetabular oncological lesions. Bone Joint J 2018; 100-B(Suppl. A): 22–30. 29292336 10.1302/0301-620X.100B1.BJJ-2017-0548.R1 PMC6424434

[4] Jansen JA van de Sande MA Dijkstra PD . Poor long-term clinical results of saddle prosthesis after resection of periacetabular tumors. Clin Orthop Relat Res 2013; 471: 324–331. 23054524 10.1007/s11999-012-2631-x PMC3528941

[5] Danışman M Mermerkaya MU Bekmez Ş , et al. Reconstruction of periacetabular tumours with saddle prosthesis or custom-made prosthesis, functional results and complications. Hip Int 2016; 26: e14–e18. 10.5301/hipint.5000306 26868113

[6] Bus MP Szafranski A Sellevold S , et al. LUMiC^®^ endoprosthetic reconstruction after periacetabular tumor resection: short-term results. Clin Orthop Relat Res 2017; 475: 686–695. 27020434 10.1007/s11999-016-4805-4 PMC5289170

[7] Issa SP Biau D Babinet A , et al. Pelvic reconstructions following peri-acetabular bone tumour resections using a cementless ice-cream cone prosthesis with dual mobility cup. Int Orthop 2018; 42: 1987–1997. 29460155 10.1007/s00264-018-3785-2

[8] Hipfl C Stihsen C Puchner SE , et al. Pelvic reconstruction following resection of malignant bone tumours using a stemmed acetabular pedestal cup. Bone Joint J 2017; 99-B: 841–848. 28566407 10.1302/0301-620X.99B6.BJJ-2016-0944.R1

[9] Guo W Li D Tang X , et al. Reconstruction with modular hemipelvic prostheses for periacetabular tumor. Clin Orthop Relat Res 2007; 461: 180–188. 17452921 10.1097/BLO.0b013e31806165d5

[10] Ogura K Susa M Morioka H , et al. Reconstruction using a constrained-type hip tumor prosthesis after resection of malignant periacetabular tumors: a study by the Japanese Musculoskeletal Oncology Group (JMOG). J Surg Oncol 2018; 117: 1455–1463. 29473959 10.1002/jso.25005

[11] Zang J Guo W Yang Y , et al. Reconstruction of the hemipelvis with a modular prosthesis after resection of a primary malignant peri-acetabular tumour involving the sacroiliac joint. Bone Joint J 2014; 96-B: 399–405. 24589799 10.1302/0301-620X.96B3.32387

[12] Liang H Ji T Zhang Y , et al. Reconstruction with 3D-printed pelvic endoprostheses after resection of a pelvic tumour. Bone Joint J 2017; 99-B: 267–275. 28148672 10.1302/0301-620X.99B2.BJJ-2016-0654.R1

[13] Wang J Min L Lu M , et al. What are the complications of three-dimensionally printed, custom-made, integrative hemipelvic endoprostheses in patients with primary malignancies involving the acetabulum, and what is the function of these patients? Clin Orthop Relat Res 2020; 478: 2487–2501. 32420722 10.1097/CORR.0000000000001297 PMC7594920

[14] Jaiswal PK Aston WJ Grimer RJ , et al. Peri-acetabular resection and endoprosthetic reconstruction for tumours of the acetabulum. J Bone Joint Surg Br 2008; 90-B: 1222–1227. 10.1302/0301-620X.90B9.20758 18757964

[15] Abudu A Grimer RJ Cannon SR , et al. Reconstruction of the hemipelvis after the excision of malignant tumours. J Bone Joint Surg Br 1997; 79: 773–779. 9331034 10.1302/0301-620x.79b5.6749

[16] Ozaki T Hoffmann C Hillmann A , et al. Implantation of hemipelvic prosthesis after resection of sarcoma. Clin Orthop Relat Res 2002; 396: 197–205. 10.1097/00003086-200203000-00030 11859244

[17] Peng W Zheng R Wang H , et al. Reconstruction of bony defects after tumor resection with 3D-printed anatomically conforming pelvic prostheses through a novel treatment strategy. BioMed Res Int 2020; 2020: 8513070. 33335928 10.1155/2020/8513070 PMC7723494

[18] Angelini A Kotrych D Trovarelli G , et al. Analysis of principles inspiring design of three-dimensional-printed custom-made prostheses in two referral centres. Int Orthop 2020; 44: 829–837. 32170471 10.1007/s00264-020-04523-y

[19] Chan LW Imanishi J Ngan SY , et al. Extracorporeal irradiation and reimplantation with total hip arthroplasty for periacetabular pelvic resections: a review of 9 cases. Sarcoma 2016; 2016: 2549616. 27199613 10.1155/2016/2549616 PMC4854988

[20] Krieg AH Mani M Speth BM , et al. Extracorporeal irradiation for pelvic reconstruction in Ewing’s sarcoma. J Bone Joint Surg Br 2009; 91; 395–400. 19258619 10.1302/0301-620X.91B3.21164

[21] Wafa H Grimer RJ Jeys L , et al. The use of extracorporeally irradiated autografts in pelvic reconstruction following tumour resection. Bone Joint J 2014; 96-B: 1404–1410. 25274929 10.1302/0301-620X.96B10.33470

[22] Jones CW Shatrov J Jagiello JM , et al. Clinical, functional and radiological outcomes of extracorporeal irradiation in limb salvage surgery for bone tumours. Bone Joint J 2017; 99-B: 1681–1688. 29212693 10.1302/0301-620X.99B12.BJJ-2016-0462.R2

[23] Hu YC Huang HC Lun DX , et al. Resection hip arthroplasty as a feasible surgical procedure for periacetabular tumors of the pelvis. Eur J Surg Oncol 2012; 38: 692–699. 22632849 10.1016/j.ejso.2012.04.014

[24] Schwartz AJ Kiatisevi P Eilber FC , et al. The Friedman-Eilber resection arthroplasty of the pelvis. Clin Orthop Relat Res 2009; 467: 2825–2830. 19384561 10.1007/s11999-009-0844-4 PMC2758972

[25] Fuchs B O’Connor MI Kaufman KR , et al. Iliofemoral arthrodesis and pseudarthrosis: a long-term functional outcome evaluation. Clin Orthop Relat Res 2002; 397: 29–35. 10.1097/00003086-200204000-00005 11953592

[26] Wang B Hao Y Pu F , et al. Computer-aided designed, three dimensional-printed hemipelvic prosthesis for peri-acetabular malignant bone tumour. Int Orthop 2018; 42: 687–694. 28956108 10.1007/s00264-017-3645-5

[27] Wong KC Kumta SM Geel NV , et al. One-step reconstruction with a 3D-printed, biomechanically evaluated custom implant after complex pelvic tumor resection. Comput Aided Surg 2015; 20: 14–23. 26290317 10.3109/10929088.2015.1076039

[28] Wang J Min L Lu M , et al. Three-dimensional-printed custom-made hemipelvic endoprosthesis for primary malignancies involving acetabulum: the design solution and surgical techniques. J Orthop Surg Res 2019; 14: 389. 31775805 10.1186/s13018-019-1455-8 PMC6882053

[29] Han Q Zhang K Zhang Y , et al. Individual resection and reconstruction of pelvic tumor with three-dimensional printed customized hemi-pelvic prosthesis: a case report. Medicine (Baltimore) 2019; 98: e16658. 10.1097/MD.0000000000016658 PMC6738983 31490360

[30] von Elm E Altman DG Egger M , et al. The Strengthening the Reporting of Observational Studies in Epidemiology (STROBE) statement: guidelines for reporting observational studies. Lancet 2007; 370: 1453–1457. 18064739 10.1016/S0140-6736(07)61602-X

[31] Henderson ER O’Connor MI Ruggieri P , et al. Classification of failure of limb salvage after reconstructive surgery for bone tumours: a modified system including biological and expandable reconstructions. Bone Joint J 2014; 96-B: 1436–1440. 25371453 10.1302/0301-620X.96B11.34747

[32] Angelini A Trovarelli G Berizzi A , et al. Three-dimension-printed custom-made prosthetic reconstructions: from revision surgery to oncologic reconstructions. Int Orthop 2019; 43: 123–132. 30467646 10.1007/s00264-018-4232-0

[33] Bus MP Boerhout EJ Bramer JM , et al. Clinical outcome of pedestal cup endoprosthetic reconstruction after resection of a peri-acetabular tumour. Bone Joint J 2014; 96-B: 1706–1712. 25452377 10.1302/0301-620X.96B12.34622

[34] Ji T Guo W Yang RL , et al. Modular hemipelvic endoprosthesis reconstruction–experience in 100 patients with mid-term follow-up results. Eur J Surg Oncol 2013; 39: 53–60. 23131428 10.1016/j.ejso.2012.10.002

[35] Wang B Xie X Yin J , et al. Reconstruction with modular hemipelvic endoprosthesis after pelvic tumor resection: a report of 50 consecutive cases. PLoS One 2015; 10: e0127263. 10.1371/journal.pone.0127263 PMC4444202 26011448

[36] Menendez LR Ahlmann ER Falkinstein Y , et al. Periacetabular reconstruction with a new endoprosthesis. Clin Orthop Relat Res 2009; 467: 2831–2837. 19693634 10.1007/s11999-009-1043-z PMC2758957

[37] Witte D Bernd L Bruns J , et al. Limb-salvage reconstruction with MUTARS^®^ hemipelvic endoprosthesis: a prospective multicenter study. Eur J Surg Oncol 2009; 35: 1318–1325. 19477098 10.1016/j.ejso.2009.04.011

[38] Müller P Dürr H Wegener B , et al. Internal hemipelvectomy and reconstruction with a megaprosthesis. Int Orthop 2002; 26: 76–79. 12078881 10.1007/s00264-001-0322-4 PMC3620871

[39] Tunn PU Fehlberg S Andreou D , et al. Endoprosthesis in the operative treatment of bone tumours of the pelvis. Z Orthop Unfall 2007; 145: 753–759. 18072042 10.1055/s-2007-965757

[40] Dai KR Yan MN Zhu ZA , et al. Computer-aided custom-made hemipelvic prosthesis used in extensive pelvic lesions. J Arthroplasty 2007; 22: 981–986. 17920469 10.1016/j.arth.2007.05.002

[41] Janssen SJ van Rein EA Paulino Pereira NR , et al. The discrepancy between patient and clinician reported function in extremity bone metastases. Sarcoma 2016; 2016: 1014248. 27725792 10.1155/2016/1014248 PMC5048023

[42] De Angelis R Sant M Coleman MP , et al. Cancer survival in Europe 1999–2007 by country and age: results of EUROCARE-5—a population-based study. Lancet Oncol 2014; 15: 23–34. 24314615 10.1016/S1470-2045(13)70546-1

[43] Pijls BG Nieuwenhuijse MJ Fiocco M , et al. Early proximal migration of cups is associated with late revision in THA: a systematic review and meta-analysis of 26 RSA studies and 49 survival studies. Acta Orthop 2012; 83: 583–591. 23126575 10.3109/17453674.2012.745353 PMC3555453

[44] Nieuwenhuijse MJ Valstar ER Kaptein BL , et al. Good diagnostic performance of early migration as a predictor of late aseptic loosening of acetabular cups: results from ten years of follow-up with Roentgen stereophotogrammetric analysis (RSA). J Bone Joint Surg Am 2012; 94: 874–880. 22617914 10.2106/JBJS.K.00305

